# Prediabetes is associated with poor functional outcome in patients with intracerebral hemorrhage

**DOI:** 10.1002/brb3.1530

**Published:** 2020-02-17

**Authors:** Qiongzhang Wang, Guiqian Huang, Fei Chen, Pinglang Hu, Wenwei Ren, Xiaoqian Luan, ChengYe Zhou, Jincai He

**Affiliations:** ^1^ Department of Neurology The First Affiliated Hospital of Wenzhou Medical University Wenzhou Zhejiang China; ^2^ Department of Neurology The Deqing People's Hospital Huzhou Zhejiang China

**Keywords:** diabetes mellitus, functional outcome, intracerebral hemorrhage, prediabetes

## Abstract

**Introduction:**

The association between prediabetes and functional outcome in cerebrovascular diseases is controversial. No study has explored the relationship between prediabetes and functional outcome in intracerebral hemorrhage patients. Our study aimed to explore the association between prediabetes and functional outcome in intracerebral hemorrhage patients 1 month poststroke.

**Methods:**

One hundred and fifty intracerebral hemorrhage patients were consecutively recruited within the first 24 hr after admission and were followed up for 1 month. Patients were divided into a diabetes mellitus group, a prediabetes group, and a nondiabetic group by fasting glucose levels, 2‐hr postprandial blood glucose levels, and glycosylated hemoglobin levels. Patients with modified Rankin Scale scores >2 at 1 month were defined as having a poor functional outcome.

**Results:**

The prediabetes group had a higher risk of poor functional outcome than the nondiabetic group in intracerebral hemorrhage patients (37.9% vs. 9.8%, χ^2^ = 11.521, *p* = .001). According to the logistic regression analyses, prediabetes was associated with a poor functional outcome in intracerebral hemorrhage patients after adjusting for confounding factors (odds ratio = 6.167, 95% confidence interval = 1.403–27.102, *p* = .016).

**Conclusions:**

Our findings show that prediabetes is associated with a poor functional outcome in intracerebral hemorrhage patients 1 month poststroke.

## Graphical abstract

This is the first study exploring the association between PreDM and functional outcome among intracerebral hemorrhage patients. We found the PreDM was associated with poor functional outcome among intracerebral hemorrhage patients. PreDM with a factor group and PreDM with multiple factors group both had higher risk of poor functional outcome than non‐DM patient group.

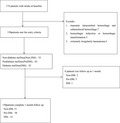

## 1 INTRODUCTION

Stroke, a common cerebrovascular disease, has been a concern for its high disability rate, high fatality rate, and serious social harmfulness (Feigin et al., 2014). The stroke burden in China has increased over the past 30 years (Wang et al., 2017). Acute ischemic stroke and intracerebral hemorrhage account for approximately 70% and 20% of all stroke, respectively (Wang et al., 2017). Chinese populations have a higher proportion of intracerebral hemorrhage than white populations (Tsai, Jeng, Anderson, & Sudlow, 2017). Despite the well‐documented effectiveness of early rehabilitation for acute stroke, the majority of stroke survivors still suffer from physical impairment, which may lead to a poor quality of life (Wolfe et al., 2011). Therefore, early prediction of functional outcome in acute stroke patients is of crucial clinical significance to achieve optimal treatment outcome.

Prediabetes (PreDM), a state between normal glucose metabolism and diabetes (DM), is characterized by impaired fasting glucose (IFG) and/or impaired glucose tolerance (IGT) and/or abnormal glycosylated hemoglobin (HbA1c) levels (American Diabetes Association, 2014). Previous studies have shown that PreDM is a strong risk factor for DM. Approximately 5%–10% of PreDM cases will progress to DM annually (American Diabetes Association, 2014; Tabak, Herder, Rathmann, Brunner, & Kivimaki, 2012). The high prevalence of PreDM during the acute period of stroke can be transient or persistent, representing undiagnosed abnormal glucose metabolism (Fonville, Zandbergen, Koudstaal, & den Hertog, 2014). A previous study has suggested that both poor control and extremely intensive diabetes control might be associated with an increased risk of intracerebral hemorrhage (Saliba et al., 2019). Whether PreDM increases the risk of intracerebral hemorrhage is still unknown. Nineteen case–control studies involving 3,397 people with ICH and 5,747 people without ICH found an association between DM and ICH occurrence, which suggested that there may be modest associations between DM and ICH occurrence and outcome (Boulanger, Poon, Wild, & Salman, 2016). Therefore, we hypothesized that PreDM might be related to functional outcome in intracerebral hemorrhage patients. In this study, we aimed to explore the association between PreDM and functional outcome in intracerebral hemorrhage patients.

## 2 METHODS

### 2.1 Study population

Patients with intracerebral hemorrhage were sent to the emergency center of our hospital. Brain CT scans were performed in all patients by professional radiologists at admission. Patients with unstable vital signs were transferred to the intensive care unit. Patients with surgical indications and whose families agreed to the operation were transferred to neurosurgery for surgery. The remaining patients were hospitalized in the stroke unit. Patients with the spontaneous intracerebral hemorrhage who were consecutively admitted to the stroke unit of our hospital between April 2014 and June 2017 were screened for study entry. This study obtained the approval of the Medical Ethics Committee of the First Affiliated Hospital of Wenzhou Medical University. All subjects gave signed informed consent to participate in the study, which was approved by the Institutional Review Board (IRB) of The First Affiliated Hospital of Wenzhou Medical University. The clinical research described in the manuscript was carried out in accordance with the Declaration of Helsinki, promulgated by the National Institute of Health. The inclusive criteria were as follows: (a) age 18–80 years; (b) intracerebral hemorrhage occurring within 7 days of entering the stroke unit; (c) diagnosed by computerized tomography (CT); (d) the ability and willingness to provide follow‐up information; and (e) complete information including fasting blood glucose, postprandial blood glucose 2 hr after an oral glucose tolerance test (OGTT) and HbA1c. We excluded patients who met the following criteria: (a) traumatic intracerebral hemorrhage and subarachnoid hemorrhage; (b) hemorrhagic infarction or hemorrhagic transformation; and (c) extremely irregularity hematoma.

### 2.2 Clinical variables

All data for this study were collected with standardized questionnaires, which included questions regarding demographic data (age, sex, etc.), lifestyle characteristics (smoking status, alcohol intake, etc.), and health status (hypertension, hyperlipidemia, coronary artery disease, history of stroke, etc.), and the time from stroke onset to admission, by trained physicians three to five days after admission. Brain CT scans were performed in all patients by professional radiologists at admission. Brain CT scans were performed again 7 days after onset in hemorrhagic stroke patients. The images were viewed, and the imaging reports were written by an imaging physician specializing in CT. The volume of lesion and hematoma in hemorrhagic stroke patients was calculated using the 2/3Sh formula at admission (Yan et al., 2013; Zhao et al., 2010). The volume of edema was calculated by subtracting the volume of the lesion from that of the hematoma. Hematoma expansion was defined as an increase in the absolute baseline volume of hematoma by either 33% or >12.5 ml on the brain CT 7 days after stroke onset (Mayer et al., 2005). Edema extension distance (EED), a recently proposed novel edema metric, was used in the primary analysis. The EED represented the average thickness in centimeters of the edema around the intracerebral hemorrhage and was calculated using the following equation: (3 (Edema volume + intracerebral hemorrhage volume) /4π) 1/3‐ (3(intracerebral hemorrhage volume)/4π) 1/3 (Parry‐Jones et al., 2015).

### 2.3 Definition of prediabetes

Blood samples were obtained from all patients within 24 hr of admission. Fasting plasma glucose levels were measured by the hexokinase (HK) method on the first morning after admission after a 12‐hr overnight fast and resting period. HbA1c levels were measured by ion‐exchange chromatography on the first morning after admission after a 12‐hr overnight fast and resting period. Postprandial blood glucose levels were measured by the HK method 2 hr after an OGTT (Hagura, 2005). Patients were divided into three groups: a DM group, a PreDM group, and a non‐DM group. DM was defined as a fasting plasma glucose level ≥7.0 mmol/L, a postprandial blood glucose 2 hr after an OGTT ≥11.1 mmol/L, an HbA1c level ≥6.5%, or a history of insulin or oral antidiabetic agent use. PreDM was defined as having impaired fasting glucose (IFG) (5.6 mmol/L to 6.9 mmol/L) or impaired glucose tolerance (IGT) (2 hr postprandial blood glucose levels, 7.8 mmol/L to 11.0 mmol/L) and/or disturbed HbA1c levels (5.7%–6.4%) (American Diabetes Association, 2014). Non‐DM was defined as fasting plasma glucose levels <5.6 mmol/L, HbA1c levels <5.7%, and 2 hr postprandial blood glucose levels <7.8 mmol/L. In addition, PreDM with a single factor was defined as having only one of the following factors: IFG, IGT, and disturbed HbA1c levels. PreDM with multiple factors was defined as having two or more of the following factors: IFG, IGT, and disturbed HbA1c levels.

### 2.4 Scale measurement

The severity of stroke was assessed by the National Institutes of Health Stroke Scale (NIHSS) at admission. The functional outcome was measured by the modified Rankin Scale (mRS) at the 1‐month follow‐up. Patients with mRS scores >2 were defined as having poor functional outcomes. All of the measurements were performed by the researchers, who were blinded to the laboratory results.

### 2.5 Statistical analysis

Results were expressed as a number (percentages) for categorical variables and as means (standard deviation, *SD*) or medians (interquartile range) for continuous variables, depending on the normal or nonnormal distribution of the data. The differences among three groups were tested by one‐way analysis of variance (ANOVA) or the Kruskal–Wallis H test for continuous variables, as appropriate. When there were significant differences among the three groups, the Bonferroni test was used to assess differences in two‐group comparisons. The differences between two groups were compared by Student's *t* test and Mann–Whitney *U* test as appropriate. Categorical variables were compared using the chi‐square test. Missing data were addressed by case deletion. The role of PreDM in functional outcome 1 month poststroke among intracerebral hemorrhage patients was evaluated by logistic regression analysis after adjusting for the variables related to functional outcome in the univariate analyses. The results were expressed as adjusted odds ratios (ORs) with the corresponding 95% confidence intervals (CIs). All statistical tests were performed with SPSS for Windows (Release 19.0; SPSS). *p* < .05 was considered statistically significant.

## 3 RESULTS

**Figure 1:**
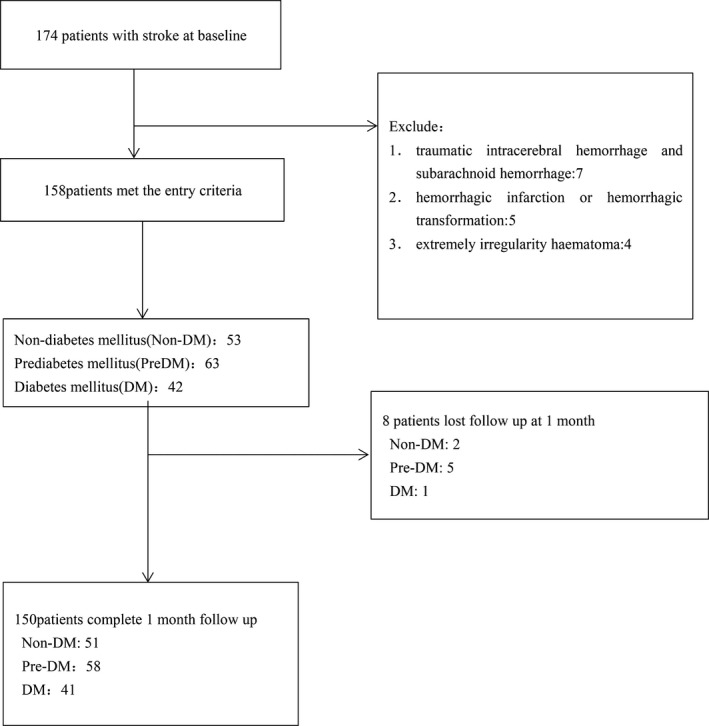
Study recruitment profile. DM, diabetes mellitus; PreDM, prediabetes

In this study, a total of 174 patients admitted with acute stroke were screened, and 158 met the entry criteria (Figure 1). By the time of follow‐up of 1 month poststroke, there were 150 patients included in our study, and 8 (5.1%) patients dropped out of this study. The excluded patients had lower NIHSS scores than the included patients (2(1–2) vs. 4(2–8), Z = −2.417, *p* = .016). There were no differences in age, sex, or glycemic status between the excluded patients and the included patients (age: 53 ± 15 vs. 61 ± 12, t = 0.578, *p* = .06; sex: 2(25%) vs. 51(34%), χ^2^ = 0.274, *p* = .601; glycemic status (DM/PreDM/non‐DM): 25.0%/62.5%/12.5% vs. 34.0%/38.7%/27.3%, χ^2^ = 0.043, *p* = .836).

There were 150 intracerebral hemorrhage patients consisting of 51 (36.8%) females, and the average age was 61 ± 12 years. There were 123 (82%) patients who were sent to the emergency center of our hospital within 24 hr. There was a significant difference in the risk of poor functional outcome among the non‐DM, PreDM, and DM groups (9.8% vs., 37.9% vs. 51.2%, respectively, χ^2^ = 19.444, *p* < .001). The risk of poor functional outcome in the PreDM group was higher than in the non‐DM group among intracerebral hemorrhage patients (37.9% vs. 9.8%, χ^2^ = 11.521, *p* = .001) (Table 1).

**Table 1:** Baseline clinical characteristics and outcome data in nondiabetic, prediabetic, and diabetic patients with intracerebral hemorrhage

	Non‐DM（*n* = 51）	PreDM (*n* = 58)	DM (*n* = 41)	*p*
Age (years), median (q1, q3)	58 ± 12	61 ± 12	65 ± 12^a^	.017
Female sex, *n* (%)	13 (25.5)	22 (37.9)	16 (39.0)	.286
Risk factors, *n* (%)				
Hypertension	33 (64.7)	44 (75.9)	31 (75.6)	.361
Hyperlipidemia	0 (0)	11 (19.0)^b^	11 (26.8)^a^	.001
CAD	0 (0)	0 (0)^c^	4 (9.8)^a^	.004
History of stroke	6 (11.8)	8 (13.8)	3 (7.3)	.601
Current smoking	21 (41.2)	21 (36.2)	15 (36.6)	.847
Current drinking	23 (45.1)	19 (32.8)	17 (41.5)	.399
Lesion location, *n* (%)				.885
Brain lobe	4 (7.8)	5 (8.6)	3 (7.3)	
Basal ganglia	25 (49.0)	23 (39.7)	20 (48.8)	
Thalamus	7 (13.7)	12 (20.7)	6 (14.6)	
Brainstem	5 (9.8)	5 (8.6)	5 (12.2)	
Cerebellum	4 (7.8)	6 (10.3)	4 (9.8)	
Other	6 (11.8)	7 (12.1)	3 (7.3)	
Hematoma volume	4.41 (1.33–11.75)	7.40 (4.30–16.78)^b^	5.63 (2.57–14.72)	.022
Hematoma enlargement	14 (27.5)	20 (34.5)	15 (36.6)	.605
Edema volume	6.88 (2.45–12.16)	11.1 (3.47–26.21)	7.31 (3.79–22.96)	.104
Edema extension distance	0.72 (−0.24–1.71)	0.82 (0.30–1.58)	0.87 (0.02–1.53)	.582
Time from stroke onset to admission (hours)	7 (3–24)	8.5 (3–24)	8 (3–48)	.716
NIHSS scores, *n* (%)	3 (1–6)	4 (2–9)	7 (2–10)^a^	.037
mRS scores ＞2, *n*(%)	5 (9.8)	22 (37.9)^b^	21 (51.2)^a^	＜.001

Note

Values are shown as number (percentage) or as median (quartiles).

Abbreviation: CAD, coronary artery disease; DM, diabetes mellitus; EED, edema extension distance; IQR, interquartile range; mRS, Modified Rankin Scale; NIHSS, National Institutes of Health Stroke Scale; PreDM, prediabetes.

a *p* < .05 for comparison between DM and non‐DM.

b *p* < .05 for comparison between PreDM and non‐DM.

c *p* < .05 for comparison between PreDM and DM.

**Figure 2:**
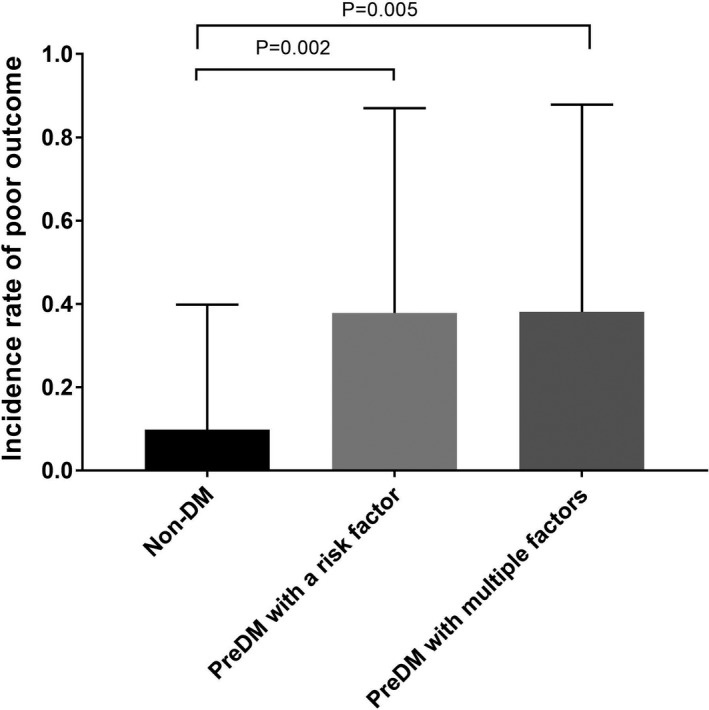
The occurrence rate of poor functional outcome among non‐DM group, PreDM with a risk factor group, and PreDM with multiple factors. DM, diabetes mellitus; PreDM, prediabetes

The PreDM group was divided into two subgroups: PreDM with a single factor and PreDM with multiple factors. There were 37 and 21 patients in the PreDM with a single factor group and the PreDM with multiple factors group, respectively. There was a significant difference in the risk of poor functional outcome among the non‐DM group, PreDM with a single factor group, and PreDM with multiple factors group (9.8% vs., 37.8% vs., 38.1%, respectively, χ^2^ = 11.522, *p* = .003). The PreDM with a single factor group had a higher risk of poor functional outcome than the non‐DM group (37.8% vs., 9.8%, χ^2^ = 9.954, *p* = .002). The PreDM with multiple factors group also had a higher risk of poor functional outcome than the non‐DM group (38.1% vs., 9.8%, χ^2^ = 8.047, *p* = .005). However, there was no difference in the risk of poor functional outcome between the PreDM with a single factor group and the PreDM with multiple factors group (37.8% vs. 38.1%, χ^2^ = 0.000, *p* = .958) (Figure 2).

The associations between potential risk factors and functional outcome in intracerebral hemorrhage patients are described in Table 2. Poor functional outcome is related to age, hyperlipidemia, hematoma volume and edema volume, hematoma enlargement, glycemic status and NIHSS scores (all *p* < .05). In the logistic regression analysis, PreDM was associated with a poor functional outcome after adjusting for possible variables (OR = 6.167, 95% CI = 1.403–27.102, *p* = .016). In addition, a large hematoma volume, hematoma enlargement, high NIHSS scores and DM were associated with a poor functional outcome (OR = 1.202, 95% CI = 1.072–1.348, *p* = .002; OR = 3.851, 95% CI = 1.286–11.530, *p* = .016; OR = 1.281, 95% CI = 1.144–1.434, *p* < .001; OR = 8.605, 95%CI = 1.774–41.747, *p* = .08) (Table 3).

**Table 2:** Baseline clinical characteristics in intracerebral hemorrhage patients with good and poor functional outcome

	Good outcome (*n* = 102)	Poor outcome (*n* = 48)	*p* value
Age (years), median (q1, q3)	59 ± 11	65 ± 13	.009
Female sex, *n* (%)	30 (29.4)	21 (43.8)	.084
Risk factors, *n* (%)			
Hypertension	71 (69.6)	37 (77.1)	.342
Hyperlipidemia	11 (10.8)	11 (22.9)	.050
CAD	2 (2.0)	2 (4.2)	.434
History of stroke	11 (10.8)	6 (12.5)	.757
Current smoking	43 (42.2)	14 (29.2)	.126
Current drinking	40 (39.2)	19 (39.6)	.966
Lesion location, *n* (%)			.970
Brain lobe	9 (8.8)	3 (6.3)	
Basal ganglia	43 (42.2)	25 (52.1)	
Thalamus	19 (18.6)	6 (12.5)	
Brainstem	11 (10.8)	4 (8.3)	
Cerebellum	11 (10.8)	3 (6.3)	
Other	9 (8.8)	7 (14.5)	
Lesion area (ml) median (q1, q3)			
Hematoma volume	4.27 (1.47–8.29)	14.72 (6.99–23.33)	<.001
Edema volume	5.60 (1.77–11.88)	20.63 (7.34–36.18)	<.001
Hematoma enlargement	28 (27.5)	21 (43.8)	.047
Edema extension distance	0.79 (0.06–1.73)	0.77 (0.39–1.50)	.672
Time from stroke onset to admission (hr)	9.5 (3–24)	7.5 (3–24)	.727
NIHSS	3（1–5）	10 (6–13)	<.001
Glycemic status			<.001
Non‐DM	46 (45.1)	5 (10.4)	
PreDM	36 (35.3)	22 (45.8)	
DM	20 (19.6)	21 (43.8)	

Note

Values are shown as number (percentage) or as median (quartiles).

Abbreviation: CAD, coronary artery disease; DM, diabetes mellitus; IQR, interquartile range; PreDM, prediabetes; NIHSS, National Institutes of Health Stroke Scale.

**Table 3:** Adjusted OR of poor functional outcome for prediabetes in patients with intracerebral hemorrhage

Variables	OR	95% CI	*p* value
Age			.302
Sex			.268
Hyperlipidemia			.308
Hematoma volume	1.202	1.072–1.348	.002
Edema volume			.375
Hematoma enlargement	3.851	1.286–11.530	.016
Time from stroke onset to admission			.219
NIHSS	1.281	1.144–1.434	<.001
Glycemic status			.021
PreDM	6.167	1.403–27.102	.016
DM	8.605	1.774–41.747	.008

Abbreviation: CI, confidence intervals; DM, diabetes mellitus; NIHSS, National Institutes of Health Stroke Scale, OR, odds ratios; PreDM, prediabetes.

## 4 DISCUSSION

To the best of our knowledge, this is the first study exploring the association between PreDM and functional outcome among intracerebral hemorrhage patients. In the present study, we found that PreDM was associated with poor functional outcome among intracerebral hemorrhage patients. Our findings suggest that it is necessary for a clinical physician to reduce the PreDM patient's glucose levels to normal after intracerebral hemorrhage.

In our study, we observed that 38.7% of intracerebral hemorrhage patients were defined as PreDM at admission, which is consistent with the occurrence rate of PreDM among ischemic stroke patients and the general population (Mijajlovic, Aleksic, Sternic, Mirkovic, & Bornstein, 2017; Vijayakumar et al., 2019). We found that the PreDM group had a higher risk of poor functional outcome than the non‐DM group among intracerebral hemorrhage patients. After adjusting for possible factors, PreDM was still associated with poor functional outcomes among intracerebral hemorrhage patients. Many (Boulanger et al., 2016; Ferrete‐Araujo, Egea‐Guerrero, Vilches‐Arenas, Godoy, & Murillo‐Cabezas, 2015; Kojic, Burina, Hodzic, Pasic, & Sinanovic, 2009; Zhang et al., 2018) but not all (Narayan, Sivaprasad, Sushma, Sahoo, & Dutta, 2012; Q. Wang et al., 2015) studies have shown that diabetes is a predictor of poor outcomes in intracerebral hemorrhage patients. Previous studies have shown that early hyperglycemia worsens the prognosis of intracerebral hemorrhage patients (Appelboom et al., 2011; Bejot et al., 2012; Stead et al., 2010; Tapia‐Perez, Gehring, Zilke, & Schneider, 2014). Both diabetes and stress hyperglycemia may be associated with poor prognosis (Passero, Ciacci, & Ulivelli, 2003; Wang, Wang, Zhang, & Qin, 2011). A previous study argued that hyperglycemia during the acute period of intracerebral hemorrhage is a stress reaction caused by a large hematoma (Tetri, Juvela, Saloheimo, Pyhtinen, & Hillbom, 2009). However, PreDM was still associated with poor functional outcome after adjusting for hematoma volume in this study. Therefore, the relationship between PreDM and poor functional outcome might not be completely explained by the stress reaction. An elevated level of HbA1c has been shown to be a predictor of poor outcome in patients with intracerebral hemorrhage (Liu et al., 2019; Zhang et al., 2015). Even previous studies suggested that IFG, IGT and impaired glucose regulation were associated with poor functional outcome among intracerebral hemorrhage patients, respectively (Osei et al., 2017; Sun et al., 2016), although the association between PreDM and functional outcome among intracerebral hemorrhage patients has not been previously studied. Due to the high prevalence of PreDM in intracerebral hemorrhage patients and the effect of PreDM on functional outcome, it is necessary for clinical physicians to reduce the glucose levels of intracerebral hemorrhage patients with PreDM. In addition, we found that the PreDM with a single factor group and the PreDM with multiple factors group both had a higher risk of poor functional outcome than the non‐DM patient group; thus, we should begin therapy at once when intracerebral hemorrhage patients are diagnosed with PreDM at admission.

The underlying mechanism of poor functional outcome caused by PreDM is still unknown. The association of DM and poor functional outcome in patients with spontaneous intracerebral hemorrhage could be explained by the following aspects. First, it might be mediated by mechanisms such as the association between DM and the occurrence of cerebral small vessel disease (Hankey et al., 2013) and the association between hyperglycemia and ICH volume expansion (Boulanger et al., 2016). Clinical research has revealed that hyperglycemia is a risk factor for cerebral microbleeds (Caunca et al., 2016), which lead to larger lobar and deep hematoma among intracerebral hemorrhage patients (Boulouis et al., 2016). In this study, the PreDM group had a larger hematoma volume than the non‐DM group. Meanwhile, our study showed that large hematoma volume and hematoma enlargement are associated with a poor functional outcome. Therefore, we speculated that in intracerebral hemorrhage patients with PreDM, a large hematoma volume might be one of a number of possible underlying mechanisms. Second, hyperglycemia was closely related to cerebral edema and peri‐hematomal cell death, which may be one of the mechanisms by which DM leads to poor outcomes (Song et al., 2003). However, no significant difference in EED was found among the non‐DM group, PreDM group, and DM group in the study. Therefore, this cannot explain the poor functional outcome caused by PreDM. In addition, hyperglycemia itself probably results in neurotoxicity (Chiu et al., 2013; Song et al., 2003), and it exacerbates cerebral injury through oxidative stress, inflammation, and neuronal apoptosis (Cao, Du, Zhang, Yan, & Hu, 2015). Abnormal intracellular calcium recovery due to hyperglycemia has been considered to play a role in the pathophysiology of poor neurological recovery (Mechanick, 2006). Because of the limitations of the research design, the study did not explore the difference in oxidative stress, inflammation, and neuronal apoptosis between different glycemic status groups. Further studies are needed to explore the underlying mechanism.

There are several limitations in our study. First, the use of antidiabetic medicine after intracerebral hemorrhage was not recorded. Second, the level of glucose was assessed only once at admission. Third, 1 month was chosen for the assessment of functional outcome and not 3 or 6 months, which might be more appropriate for assessing functional outcome. Fourth, the sample size of our study was small. Fifth, patients with unstable vital signs and patients who chose to undergo surgery were excluded, so the hematoma volume for all included patients was relatively small.

## 5 CONCLUSIONS

In conclusion, our study demonstrates that PreDM is associated with a poor functional outcome after intracerebral hemorrhage. PreDM, which is a prevalent glycemic status in intracerebral hemorrhage patients, should be given more attention. Our findings have substantial implications for the early prediction of functional outcome and improvement of functional outcome among intracerebral hemorrhage patients. A large sample size study is needed to explore the relationship between PreDM and the long‐term functional outcome of intracerebral hemorrhage, and the underlying mechanism of our findings needs to be further explored.

## CONFLICT OF INTEREST

The authors declare that there are no conflicts of interest.

## Data Availability

This study protocol was approved by the Medical Ethics Committee of the First Affiliated Hospital of Wenzhou Medical University. All procedures performed involving human participants were in accordance with the ethical standards of the institutional and national research committee and with the 1964 Helsinki Declaration and its later amendments or comparable ethical standards. Informed consent was obtained from all individual participants included in the study. The data that support the findings of this study are available from the corresponding author upon reasonable request.
